# Bulk Milk Tank Samples Are Suitable to Assess Circulation of Tick-Borne Encephalitis Virus in High Endemic Areas

**DOI:** 10.3390/v13091772

**Published:** 2021-09-05

**Authors:** Arnoldas Pautienius, Gytis Dudas, Evelina Simkute, Juozas Grigas, Indre Zakiene, Algimantas Paulauskas, Austeja Armonaite, Dainius Zienius, Evaldas Slyzius, Arunas Stankevicius

**Affiliations:** 1 Virology Laboratory, Institute of Microbiology and Virology, Faculty of Veterinary Medicine, Lithuanian University of Health Sciences, Tilzes str. 18, 47181 Kaunas, Lithuania; juozas.grigas@lsmuni.lt; 2 Laboratory of Immunology, Department of Anatomy and Physiology, Faculty of Veterinary Medicine, Lithuanian University of Health Sciences, Tilzes str. 18, 47181 Kaunas, Lithuania; evelina.simkute@lsmuni.lt (E.S.); indre.zakiene@lsmuni.lt (I.Z.); austeja.armonaite@lsmuni.lt (A.A.); arunas.stankevicius@lsmuni.lt (A.S.); 3 Gothenburg Global Biodiversity Centre, Carl Skottsbergs gata 22B, 41319 Gothenburg, Sweden; gytisdudas@gmail.com; 4 Department of Biology, Faculty of Natural Sciences, Vytautas Magnus University, Universiteto str. 10, 53361 Kaunas, Lithuania; algimantas.paulauskas@vdu.lt; 5 Department of Veterinary Pathobiology, Faculty of Veterinary Medicine Lithuanian University of Health Sciences, Tilzes str. 18, 47181 Kaunas, Lithuania; dainius.zienius@lsmuni.lt; 6 Department of Animal Breeding, Faculty of Animal Science, Lithuanian University of Health Sciences, Tilzes str. 18, 47181 Kaunas, Lithuania; evaldas.slyzius@lsmuni.lt

**Keywords:** TBEV, TBEV in milk, alimentary TBE, TBEV prevalence, tick-borne encephalitis, flavivirus

## Abstract

A reliable surveillance strategy of tick-borne encephalitis virus (TBEV) is necessary to ensure adequate disease control measures. However, current approaches assessing geographical TBEV circulation are ineffective or have significant limitations. In this study we investigated a total of 1363 goat and 312 sheep bulk tank milk samples for the presence of TBEV. Samples were collected from systematically selected farms in Lithuania every 4–5 days from April to November in 2018 and 2019. To validate results, we additionally tested 2685 questing ticks collected in the vicinity of milk collection sites. We found 4.25% (95% CI 3.25–5.47) and 4.48% (95% CI 2.47–7.41) goat and sheep milk samples to be positive for TBEV, respectively. Furthermore, geographical distribution of TBEV in milk samples coincided with the known TBE endemic zone and was correlated with incidence of TBE in humans in 2019. When sampling time coincides, TBEV detection in milk samples is as good a method as via flagged ticks, however bulk milk samples can be easier to obtain more frequently and regularly than tick samples. The minimal infectious rate (MIR) in ticks was 0.34% (CI 95% 0.15–0.64). Therefore, our results confirm that testing milk serves as a valuable tool to investigate the spatial distribution of TBEV at higher resolution and lower cost.

## 1. Introduction

Tick-borne encephalitis virus (TBEV) is a zoonotic flavivirus that is considered to be the most important causative agent of tick-borne infections in Europe [1]. TBEV is maintained in nature by *Ixodes* ticks that serve as vectors and constitute the central reservoir for the virus [2]. Although TBEV is usually transmitted to humans through the bites of infected ticks, alimentary infection via unpasteurized milk or milk products has been recognized as an additional route of transmission. In most alimentary cases, TBEV is transmitted after consumption of goat milk, but infection through cow or sheep milk has been reported as well [3,4,5,6,7].

Targeted disease control measures need to be taken based on known virus prevalence. However, human surveillance alone is not enough to effectively monitor the circulation of TBEV, since national health authorities of European countries use non-uniform TBE case definitions and TBE risk assessment strategies [8]. Moreover, such estimations may be affected by uneven vaccination coverage [9]. Socioeconomic, political, environmental, or meteorological factors may also play a role in determining exposure risk [10,11,12,13]. 

Detection of TBEV in ticks by the flagging and dragging method is known as the common method to assess TBEV circulation [14]. However, the spatiotemporal mismatch between TBEV prevalence in ticks and clinical case notifications in humans has been reported as the main caveat associated with this method [15]. 

Milk sample testing has been suggested as a promising alternative to determine TBEV prevalence [2]. Two antibody screening investigations of milk in Sweden confirmed that it showed great applicability in mapping high-risk areas [16,17]. However, to the best of our knowledge, only two studies explicitly aiming to detect TBEV itself have been performed, both of which looking at just over a hundred samples [18,19]. 

Given the high prevalence of TBEV and the common occurrence of foodborne infections, we hypothesize that milk sample testing may help to assess the prevalence of the virus in the environment and may serve as a complement to ongoing monitoring efforts. Therefore, the overall rationale of our study was to evaluate the prevalence of the virus in the milk of small ruminants in Lithuania, where TBEV is endemic throughout the country, and to investigate whether milk sample testing can be adopted as a TBEV surveillance strategy. 

## 2. Materials and Methods

### 2.1. Sample Collection 

Goat and sheep milk samples were collected because of their known or suspected association with food-borne outbreaks, as well as favorable density and homogenous geographical distribution in Lithuania. 

The sampling frame was based on data from the National Livestock Register database of Lithuania. Farms were selected from each municipality according to a stratified random sample collection strategy and were included in the study if the following criteria were met: the owners agreed to participate in the study; animals were not vaccinated against any flaviviruses and were not acaricide-treated; farms did not apply consistent environmental tick control measures, and they were not in an urban area. 

To avoid missing viremic episodes, all milk samples were collected once every 4–5 days from bulk milk tanks throughout the lactation period between April and November in 2018 and 2019. Each sample of 10–15 mL in volume was stored frozen at −20 °C until further use. We analyzed time intervals between positive samples and measured their viral load, thus removing all consecutive positive samples attributable to a single infection.

Simultaneously, ticks were collected by the flagging and dragging method on farms or in their vicinity, in both 2018 and 2019, at a single point in time between April and November. Ticks were pooled according to development stage, sex, species, and sampling site. Up to 10 *Ixodes ricinus* adults, 20 nymphs, 50 larvae, or 5 *Dermacentor reticulatus* were grouped per pool. After collection, ticks were maintained alive until reaching the laboratory.

Data on human TBE incidence were obtained from the Centre for Communicable Diseases and AIDS of Lithuania. 

### 2.2. TBEV Detection and Viral Load Quantification 

The fat fraction of the milk was removed as described elsewhere [19]. Ticks were dissected and homogenized in liquid nitrogen and ground to a fine powder using a mortar and pestle. 

Viral RNA from skimmed milk or centrifuged tick supernatant was extracted using GeneJET RNA Purification Kit (Thermo Scientific, Waltham, MA, USA) according to the manufacturer’s instructions. Samples were tested by RT-PCR for the presence of TBEV-specific RNA using primers described previously [20]. PCR-positive samples were confirmed by partial genome sequencing targeting NCR region of TBEV using primers described before [21]. Reaction mix SuperScript™ III One-Step RT-PCR System with Platinum™ *Taq* DNA Polymerase (Thermo Scientific, Waltham, MA, USA) was used for real time PCR and DreamTaq Green PCR Master Mix (2×) (Thermo Scientific, Waltham, MA, USA) for conventional PCR. 

Viral load was determined using a quantification assay, whereby sample concentration was assessed using a calibrated standard curve derived from measurements of serial dilutions of TBE virus samples with known concentrations. All reactions were carried out in triplicate and values averaged. 

Quality assessment of RNA extraction of tick samples was performed in the same way as a previous study (20). 

### 2.3. TBEV Isolation

Vero (ATCC^®^ CCL-81^™^, Manassas, VA, USA) cells were inoculated with 300 μL aliquots of microfiltrated TBEV-RNA positive suspensions. After 1 h incubation, suspensions were discarded, cells were washed with PBS and cultured at 37 °C in 5% CO_2_ in Minimum Essential Medium with 10% heat-inactivated fetal bovine serum (FBS; Gibco, Grand Island, NY, USA) and 100 U mL^−^^1^ penicillin and 100 μg L^−^^1^ streptomycin. Cytopathic effects were examined over five serial passages and performed in triplicate for each round. The success of isolation was assessed by RT-PCR followed after RNA extraction.

## 3. Results

### 3.1. TBEV Prevalence in Milk

A total of 1363 goat and 312 sheep unpasteurized bulk milk samples taken from 17 and 4 farms, respectively, were examined for the presence of TBEV RNA.

Overall, 62 (4.54%) and 14 (4.48%) goat and sheep bulk milk samples, respectively, were confirmed positive for TBEV. However, two cases of three consecutive positive samples per farm were also identified. Based on gradual decrease in viral load we determined these cases to be two individual infections. All remaining samples appeared to be positive with a minimal interval of 10–11 days to a maximum range of 3 months. Therefore, the overall number of TBEV positive samples was adjusted to 58/1363 (4.25%, 95% CI 3.25–5.47) for goat and 14/312 (4.48%, 95% CI 2.47–7.41) for sheep samples. At least one positive sample was detected in 70.58% and 64.70% of tested goat farms in 2018 and 2019, respectively. At least one positive sample was detected in 75% of sheep farms during both years of the study.

Though sheep milk sample sizes were small, they demonstrated the same prevalence pattern seen in goat milk samples. Thus, statistical analysis was performed using only the goat samples, but also recalculated using all data. Due to low statistical power, no differences between species were identified.

**Figure 1:**
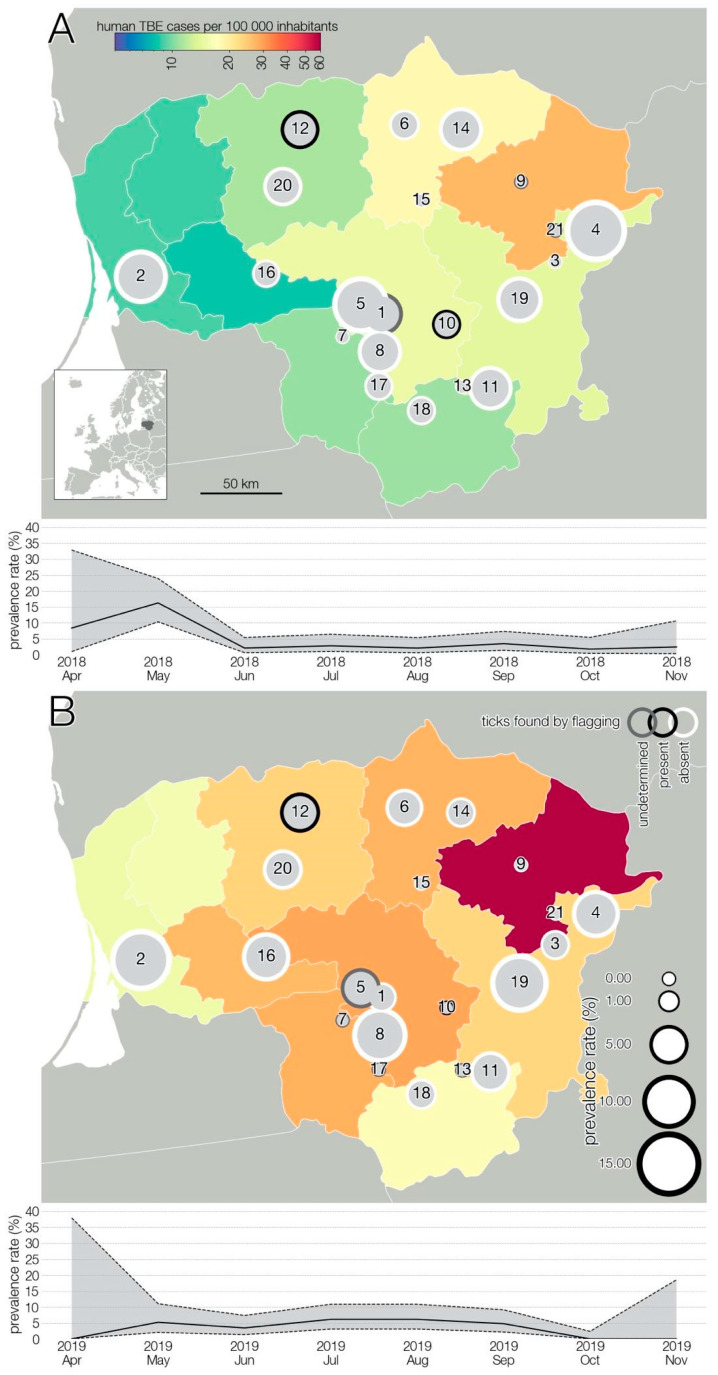
The geographic location of the study area and spatio-longitudinal distribution of TBEV positive cases in small ruminant farms. Results of 2018 (**A**), results of 2019 (**B**). The size of circles indicates TBEV prevalence rate in a given farm. Number 1–17—goat farms; 18–21—sheep farms. Colored administrative units indicate human TBE cases at NUTS3 level.

A summary of sample collection sites and TBEV spatial distribution is presented in Figure 1. To ensure privacy, geographic coordinates of each farm were randomly shifted up to 0.1° from their actual locations. Analysis of the geographic distribution shows that positive samples are fairly evenly distributed throughout the territory of Lithuania and there is no statistical association indicating that any particular area is at higher risk of TBEV. 

**Figure 2:**
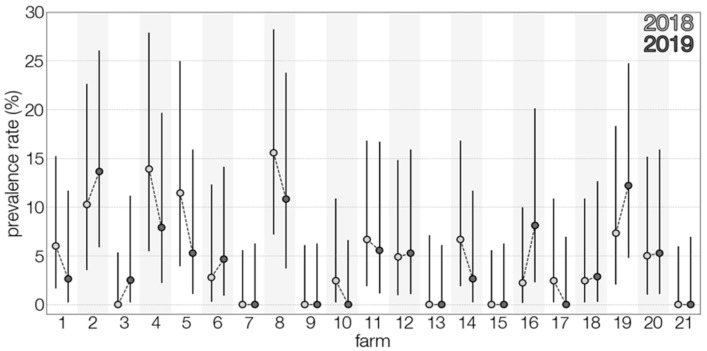
Distribution of TBEV prevalence rates amongst tested farms between the two years of study.

**Figure 3:**
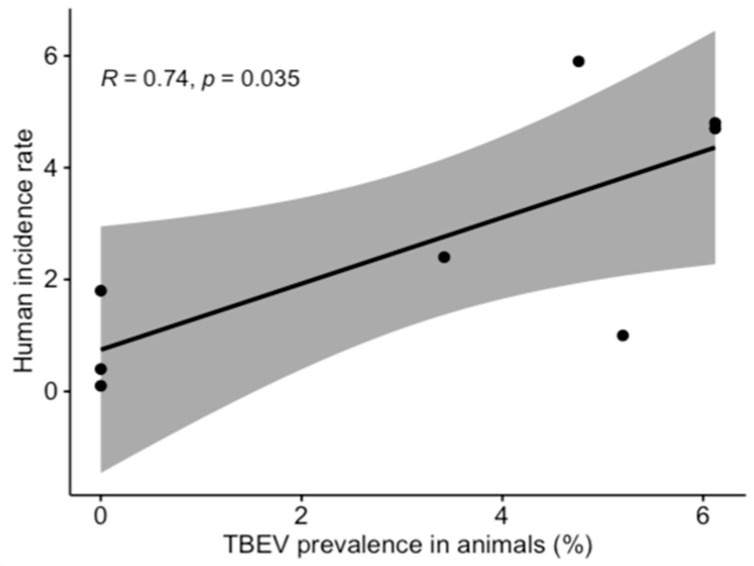
Correlation between TBEV prevalence in animals and human incidence rate (2019 data were used for analysis).

TBEV prevalence, as detected, fluctuated in time (Figure 1), and varied during the two lactation periods (Figure 2). However, limited data precluded a meaningful interpretation of the effect of time on the occurrence of positive samples. Temporal analysis showed a significant association between monthly virus prevalence in animals and TBE incidence rates in humans in 2019 (Figure 3). The same correlation in 2018 (r = 0.65) was not significant but showed a trend toward significance (*p* = 0.083). 

**Figure 4:**
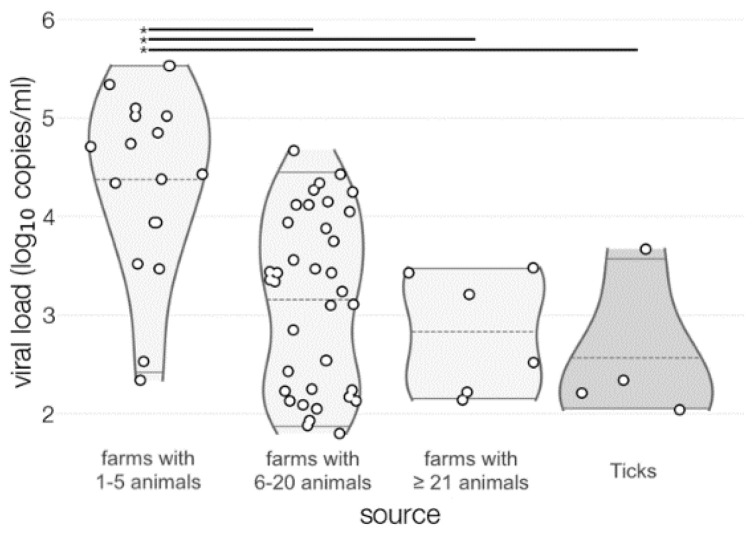
Viral load expressed as log_10_ viral RNA copies/mL. Asterisks and horizontal lines at the top of violin plots indicate statistically significant difference (*p* < 0.05) in viral load based on Tukey’s honest significance test.

A non-linear correlation was observed between TBEV prevalence and farm sizes (6–20 > 1–5 > more than 21 animals) (r = −0.54, *p* = 0.01). Moreover, a linear correlation was observed between virus load and milk amount produced in the farm (r = −0.64, *p* < 0.005) (Supplementary Material S1). Results of viral loads are presented in Figure 4. 

### 3.2. TBEV Prevalence in Ticks 

A total of 2685 questing ticks were collected corresponding to 886 adults, 1329 nymphs, and 88 larvae of *Ixodes ricinus*, and 382 *Dermacentor reticulatus* adults. No ticks were found in 3 and 4 sampling locations in 2018 and 2019, respectively.

**Figure 5:**
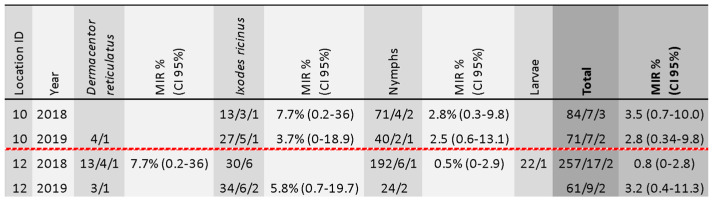
Summary of TBEV positive sample characteristics in ticks. Location ID refers to the map in Figure 1. The sequence of three numbers is explained as follows: total tick sample size/number of pools/number of positive pools.

Of the tested 283 tick pools, we found nine pools positive for TBEV-RNA, corresponding to only two collection sites in which positive samples were detected in both years of the study. Positive tick cases are summarized in Figure 5. The overall minimum infectious rate (MIR) was 0.34% (CI 95% 0.15–0.64). 

Amplification of 16S rRNA was successful in all randomly selected pools, confirming there were no false negative results due to inhibition of the PCR assay by tick-originated products. 

Specificity of both tick and milk PCR positive sample product was confirmed by partial genome sequencing based on the NCR fragment of the TBEV genome (Figure S1). Sequences of TBEV strains, including Neudörfl, U27495; Sofjin, AB062064, and Vasilchenko, AF069066 were used for phylogenetic comparisons. Analysis showed that all detected TBEV strains belong to the European subtype. Sequences have been submitted to GenBank under accessions MZ664211-MZ664256.

### 3.3. Comparison of TBEV Detection Methods

Marginal homogeneity between two TBEV surveillance approaches was assessed by McNemar’s test. Cases when ticks were not collected on site due to their scarcity were excluded from the contingency table. To compare the capacity of flagging and dragging and bulk milk methods to detect TBEV, milk samples nearest to tick samples in time were analyzed. The data provided no evidence to reject the null hypothesis, thus implying both methods are capable of detecting TBEV when sampling times coincide. 

**Figure 6:**
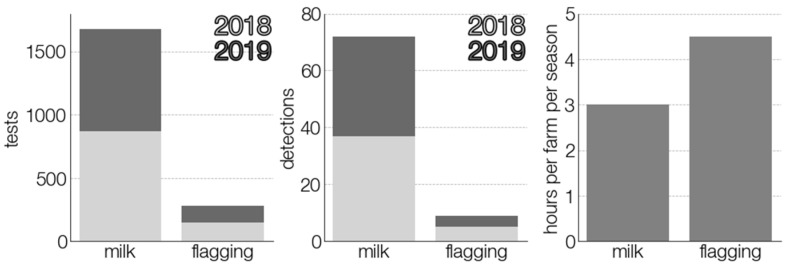
Comparison of two TBEV surveillance strategies. The time spent on sample collection is calculated assuming that it takes two and a half hours for one person to collect ticks and another two hours for tick characterization and pool formation.

However, milk sampling showed greater effectiveness in terms of time and personnel resources. By our generalized calculations, collection of one milk sample did not take more than 5 min as they were voluntarily collected by the farmers themselves. Their periodic collection and delivery to the laboratory time was also short, due to the small size of the country and optimized travel routes. A brief comparison of the two testing approaches is presented in Figure 6. Nevertheless, these results are highly country-specific and, therefore, should be judged accordingly. 

### 3.4. Virus Isolation

To confirm the presence of infective virus, all positive samples were inoculated on Vero cells that were examined for occurrence of cytopathic effect (CPE) characterized by lysis of the cell monolayer. From cultures with no visible CPE, additional sub-passages were carried out. Cells were harvested after 4–7 days. Overall, 6/9 (66%, 95% CI 29.9–92.5) tick homogenates and 13/58 (22.4%, 95% CI 12.5–35.2) goat milk suspensions were successfully isolated and caused CPE beginning 4–6 days post-infection (p.i.). Only one TBEV isolate from sheep milk was successfully isolated. Virus load varied significantly depending on passage number and showed 1–3 log_10_ increase at final passage (data not shown). 

## 4. Discussion

In this study, a novel and reliable approach for monitoring the prevalence of TBEV is presented. Herein, we conceptualized an epidemiological monitoring strategy where bulk milk tank samples were collected once every 4–5 days to include the time period when the virus is no longer shed through milk, based on previous studies showing that TBEV is detectable in milk for 3-8 days p.i. [22]. Evidence from other studies showed that TBEV may be detectable for a slightly longer period [23,24]. As our results showed, the number of positive samples could be overestimated only to a very limited extent because of the possible misclassification of multiple samples of single infection as separate cases. 

TBEV surveillance by tick flagging was carried out in parallel to the longitudinal study of milk samples. TBEV surveillance by tick flagging typically involves collecting ticks from a site once per season, whereas the proposed TBEV surveillance through longitudinal milk sample testing involved collecting approximately 80 milk samples at regular intervals during the season (April to November). The surveillance strategy based on milk sample testing proved to be more reliable as it allowed the detection of TBEV circulation over a wider geographical area, resulting in approximately two thirds of tested sites being positive, contrary to the tick flagging method where only two sites were positive for TBEV (11.4% of tested locations). Furthermore, the wide geographical distribution of TBEV in milk samples coincided with the known area of TBE endemicity, and monthly prevalence of TBEV in milk samples statistically correlated with monthly human incidence rate in one of the two investigated years (2019). The statistical association between aforementioned values was not observed in 2018 due to the remarkably high TBEV prevalence in milk in May, which in turn can possibly be explained by exceptionally favorable environmental conditions affecting the development of ticks. 

In agreement with our results, a very similar TBEV prevalence pattern in ticks and almost identical overall MIR was observed in a nationwide study conducted in Lithuania in 2017–2019 where almost 9000 ticks were tested [25]. The latter study also showed a patchy distribution of TBEV with many administrative units apparently free of the virus. These results add weight to previous findings of spatio-temporal patchiness of TBEV in ticks and discordance between numbers of clinical TBE cases in humans and prevalence in ticks [15,26,27,28,29]. 

Unfortunately, straightforward statistical comparisons of the two testing approaches were not possible due to different study designs. Data from our analysis, however, show that both methods allow us to determine the presence of the virus in the environment with reasonable accuracy when they are carried out in close proximity. Thus, it can be said that the flagging and dragging method is a sensitive method to calculate TBEV prevalence in an area but only if considerably high numbers of ticks are tested, which in turn requires unreasonably high financial and personnel resources. This is an important aspect to highlight in the context of numerous studies that have failed to detect the virus in known TBE foci, even when approximately ten or twenty thousand ticks were tested [25,26]. 

The flagging and dragging method has more drawbacks. In addition to those discussed by other authors, such as low TBEV prevalence rates, great spatiotemporal variability, and time-consuming and labor-intensive sample collection [30,31,32,33], we also want to highlight the issue of the risk of being bitten, particularly in areas where Lyme borreliosis is prevalent and against which a vaccine does not yet exist. 

TBEV ecology is advantageous to animal-based surveillance systems, as it has been amply shown that inferred TBEV prevalence based on ticks removed from hosts is higher compared to questing ticks [14,34,35]. Furthermore, an experimental study revealed that TBEV replication is faster in feeding ticks, resulting in a 500-fold increase in viral load over a 15 h observation period, while in unfed ticks it remains stable [35]. The same study indicated that infected ticks showed highest levels of activity and aggressiveness. All this suggests that animals are likely to amplify the signal of TBEV presence in a given area, which hypothetically increases the chances of successful viral detection. 

We believe that our proposed TBEV surveillance technique based on milk sample testing is robust and reliable, in addition to being in good agreement with most of CDC Guidelines for Evaluating Public Health Surveillance Systems recommendations [36]. Generally, ease of operation is one of the key features of our proposed method. This strategy does not require much coordination with numerous institutions or qualified staff. We also believe this method to be more efficient in terms of time, as sampling can rely on volunteer farmers, while the periodic collection of milk could be guaranteed by specialists from local veterinary services. The admissibility criterion, i.e., the willingness of all involved to adopt the method, was also fully met. Most farmers kindly agreed to participate in the study. Many of them showed great interest, upon learning of the investigation, and sought to be included as participants due to concerns about the quality and safety of their products. If the engagement were continuous, paid testing could be considered, especially since it would further allow extension of the flexibility criterion which cannot be considered negligible. Milk testing for TBEV can be integrated into ongoing milk-borne disease monitoring programs or regular milk quality and safety control schemes using currently available infrastructure. 

Our proposed method does not allow year-round monitoring, however the period during which small ruminants graze and produce milk coincides perfectly with the seasonal distribution of confirmed TBE cases in humans [37]. Although it appears that the density of small ruminants in Western Europe is much higher than in Lithuania [38], there seem to be some minor shortcomings in applying this method to other countries due to non-homogenous geographic dispersion of farms. In Germany, for example, the majority of small ruminant farms are situated in the southern states [32]. In such cases, the method could at least be adopted for monitoring targeted endemic areas or identifying suspected foci. 

Stability and availability are two other features that comply with CDC recommendations. Consumption of unpasteurized milk and related products appears to be on the rise due to alleged health benefits and better taste [18]. A similar trend is observed in the Baltic states. In most of the farms we surveyed, the owners consumed untreated milk products or produced them for sale. As far as farm owners should adapt to market conditions, it is unlikely that this sector will lose its potential applicability to perform a TBEV monitoring program. 

Finally, milk sample testing may offer an additional advantage, namely the assessment of milk safety. To date, there have not been many attempts to isolate TBEV from milk. Moreover, such efforts have mostly been implemented in epidemiological analyses of alimentary outbreaks rather than routine molecular screening [39]. Although accurate empirical data and detailed epidemiological studies are not available, it is thought that 7.8% of TBE cases in Lithuania are milk-borne [37]. An even higher fraction was recently observed in Slovakia where up to 17% of TBE cases are caused by alimentary transmission. The relevance of this problem was confirmed by our results showing that nearly one-fifth of positive milk samples were viable to infect cells. In light of high rates of alimentary transmission, periodic milk testing and safety assessment for TBEV, accompanied by public educational activities regarding potential risks of untreated milk product consumption, are greatly needed. 

We should also note the gap in fundamental understanding of animal immune responses against TBEV. Based on limited data, animals previously infected with TBE appear to cease shedding the virus into milk [24]. However, it is not clear whether all animals exposed to the virus develop an immune response [40,41]. While the application of our proposed method is certainly not hindered by unstable farm populations that are constantly being replenished by new susceptible animals, further background research would allow for the development of a more precise surveillance strategy. 

A number of TBEV monitoring strategies based on serological testing of various vertebrate hosts have recently been developed, many of which show promise [41,42,43,44,45,46]. However, seroprevalence studies have significant limitations. Antibody persistence rates vary, making it difficult or impossible to predict when an infection occurred. Due to well-known cross-reactivity of TBEV with related flaviviruses [47], serological assessment requires confirmatory assays, which in turn increases the need for more labor and infrastructure, while the interventional nature of studies involving domestic animals requires not only qualified staff and bioethical authorization, but also consent from farm owners, often leading to many farmers refusing to participate in this type of research due to adverse effects on animal productivity.

In conclusion, bulk milk tank samples of small ruminants may serve as a valuable tool for TBEV prevalence analysis and assessment of the epidemiological situation. Because the technique we propose is reliable, non-invasive and easy-to-operate, it may be considered for national surveillance or adapted for monitoring endemic areas and complement human TBE surveillance efforts. 

## Data Availability

The data presented in this study are available on request from the corresponding author. The data are not publicly available due to confidentiality agreements.

## Supplementary Materials

The following are available online at https://www.mdpi.com/article/10.3390/v13091772/s1, Table S1: Milk amount per bulk tank in tested farms, Figure S1: Phylogenetic tree of the obtained TBEV sequences based on NCR genome fragment.
