# Supplementary material for: Bulk Milk Tank Samples Are Suitable to Assess Circulation of Tick-Borne Encephalitis Virus in High Endemic Areas
Source: Viruses. 2021 Sep 5;13(9):1772. doi: 10.3390/v13091772 (PMC8472847; doi:10.3390/v13091772)
Supplement: Supplementary file 1 [file viruses-13-01772-s001.zip › viruses-1349386-supplementary.pdf]

Table S1. Milk amount per bulk tank in tested farms. 1–4—sheep farms; 5–21—goat farms. Data on farm ID referring to the map in Figure 1 are not provided due to personal data protection.

	Milk amount per bulk tank (liters)	Number of animals per farm	Number of TBEV positive samples	TBEV viral load (log_{10} copies/mL)		
				Mean	Min	Max
1	2.4	4	2	4.1	3.5	4.7
2	1.8	3	8	4.3	2.3	5.5
3	6	10	4	3.0	2.0	4.1
4	28.2	47	0			
5	21	15	4	2.3	1.8	3.1
6	11.2	8	10	3.7	3.2	4.3
7	56	40	1	2.2		
8	12.6	9	8	2.8	1.8	4.1
9	11.2	8	6	3.1	2.0	4.2
10	19.6	14	3	2.0	1.8	2.2
11	5.6	4	0			
12	4.2	3	7	4.4	3.4	5.5
13	77	55	0			
14	84	60	1	2.5		
15	8.4	6	5	2.6	1.9	4.1
16	28	20	4	3.0	2.4	3.3
17	224	160	0			
18	30.8	22	4	3.0	2.1	3.5
19	77	55	0			
20	8.4	6	4	4.2	3.4	4.6
21	11.2	8	1	3.1		

[1] Approximate amount of milk from one milking.

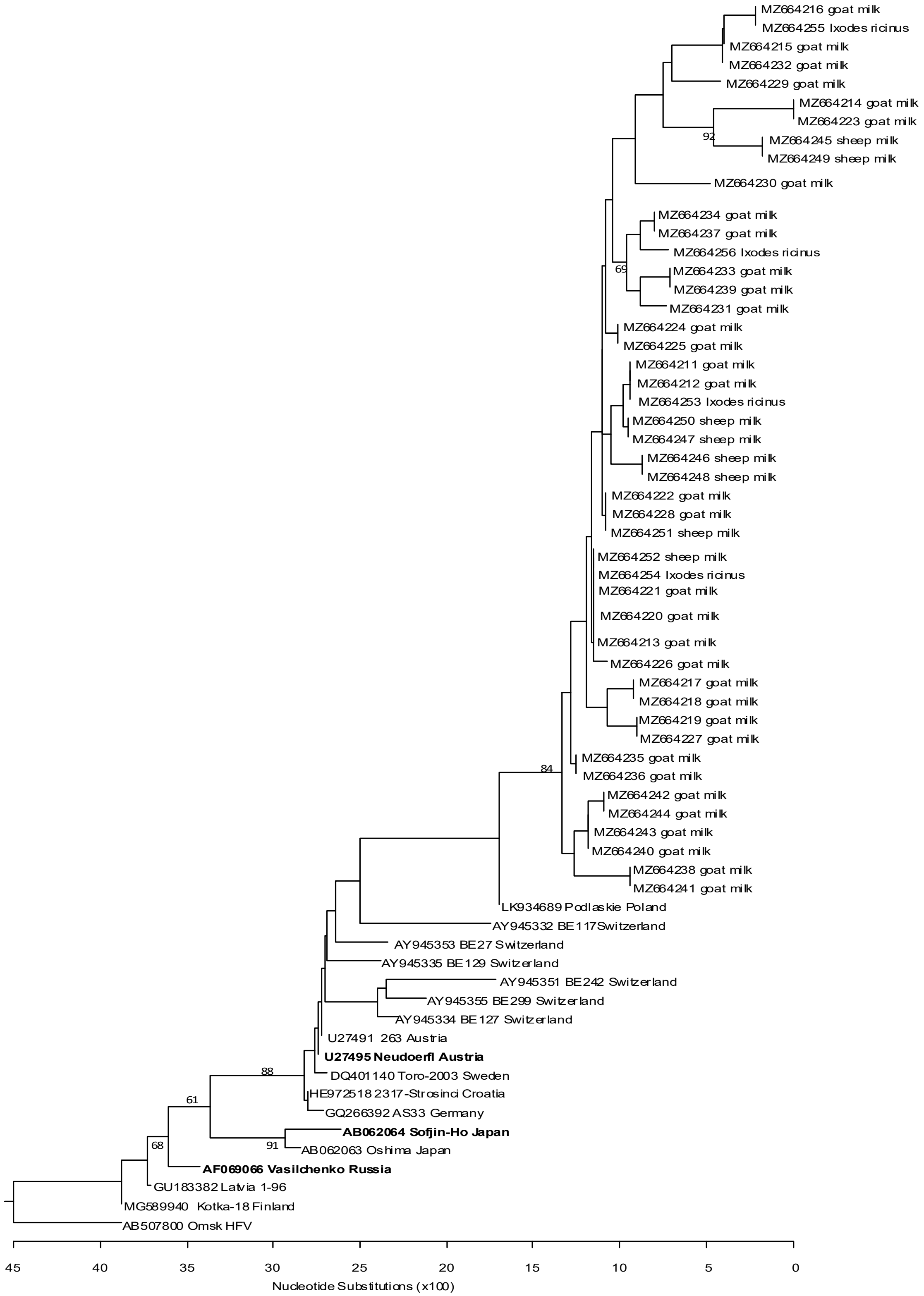

Figure. S1. Phylogenetic tree of the obtained TBEV sequences based on NCR genome fragment. The Omsk hemorrhagic fever virus was used as an outgroup. Names of the sequences indicates Accession Numbers in GenBank.
